# Bisphenol A, S or F mother’s dermal impregnation impairs offspring immune responses in a dose and sex-specific manner in mice

**DOI:** 10.1038/s41598-021-81231-6

**Published:** 2021-01-18

**Authors:** Yann Malaisé, Corinne Lencina, Christel Cartier, Maïwenn Olier, Sandrine Ménard, Laurence Guzylack-Piriou

**Affiliations:** Neuro-Gastroenterology and Nutrition, Toxalim, Université de Toulouse, INRAE, ENVT, INP-Purpan, UPS, 31027 Toulouse, France

**Keywords:** Mucosal immunology, Environmental impact

## Abstract

Bisphenol (BP)A is an endocrine disruptor (ED) widely used in thermal papers. Regulatory restrictions have been established to prevent risks for human health, leading to BPA substitution by structural analogues, like BPS and BPF. We previously demonstrated that oral perinatal exposure to BPA had long-term consequences on immune responses later in life. It appears now essential to enhance our understanding on immune impact of different routes of BP exposure. In this study, we aimed at comparing the impact of mother dermal exposure to BPs on offspring immune system at adulthood. Gravid mice were dermally exposed to BPA, BPS or BPF at 5 or 50 μg/kg of body weight (BW)/day (d) from gestation day 15 to weaning of pups at post-natal day (PND)21. In offspring, BPs dermal impregnation of mothers led to adverse effects on immune response at intestinal and systemic levels that was dependent on the BP, the dose and offspring sex. These findings provide, for the first time, results on long-term consequences of dermal perinatal BPs exposure on immune responses in offspring. This work warns that it is mandatory to consider immune markers, dose exposure as well as sex in risk assessment associated with new BPA’s alternatives.

## Introduction

Bisphenol A (BPA) and its structural analogues are widely used not only as epoxy resins for the inner coating of food and beverage cans, but also as dye in thermal papers. The European Food Safety Agency (EFSA) determined that exposure to BPA by handling thermal paper is the second largest source of exposure after oral route^1,2^. Because BPA is a well-known endocrine disruptor able to migrate from materials to food, beverage or skin, regulatory restrictions have been established to prevent risks for human health^1^. In Europe, the use of BPA at concentrations higher than 0.02% of body weight will be banned in 2020 in thermal paper^3^. Alternatives to BPA used in thermal paper are mainly other bisphenols like bisphenol S (BPS) and bisphenol F (BPF), which have few restriction so far^3–5^. Only Switzerland has decided to ban BPS in thermal paper in 2019, and companies have until June 2020 to comply with the new regulations.

Due to its higher thermal stability^6^, BPS is now widely used to replace BPA in paper products^3, 7^. The use of BPS in thermal papers may contribute to human exposure through dermal and hand-to-mouth oral exposures^8^. Indeed, the increased rate of BPS detection in urine samples collected between 2000 and 2014 (n = 616) in US adult volunteers reflects the reality of substituting BPA with BPS^9^. However, the prevalence and level of human exposure may also be increased by potential accumulation of BPS in the environment resulting from its lower biodegradability in sea water compared to BPA^10^. Even though it has a close structure compared to BPA’s structure, in vitro studies demonstrated that BPS has a lower affinity than BPA for human nuclear Estrogen Receptors (ER) α and β^11, 12^. However, BPS is able to bind to nuclear and membrane ERs, and shows weak androgen activity at very low concentrations^11, 13^. BPF, another structural analogue of BPA, is increasingly used in epoxy resins and container coatings due to its durability (i.e., high-solid/ high-build systems)^14^. Indeed, 42% to 88% of urine samples collected in the US from 2000 to 2014 contained BPF^9^. Additionally, in vitro studies showed that BPF has oestrogeno-mimetic properties similar to those of BPA^15^. Consequently, the increasing use of BPF as well as its established endocrine disruptor properties, made it also a concerning environmental contaminant^7, 16^.

In recent years, BPA regulations were more focused on the protection against exposure during fetal and neonatal periods. Indeed, BPA, BPS and BPF were detected in human placenta^17–19^ and animal studies suggested that BPA exposure during developmental stages could harm the developing immune system in the offspring and contribute to growing incidence of non-communicable diseases (NCDs) like inflammation, allergies or autoimmune diseases^20^. Epidemiological studies in humans showed that BPA exposure during prenatal and postnatal period is associated with NCDs during childhood and adulthood, with sex specific effects^21–23^. Those epidemiological studies highlighted immune system as a critical target for BPA perinatal exposure. In accordance with these observations, we previously reported that BPA exposure via oral gavage of mothers during gestation and lactation induced food intolerance and exacerbated mucosal inflammation in rodent adult offspring^24^. Similar BPA exposure via oral gavage of mothers also damaged systemic immune response and hence increased animal susceptibility to an enteric parasitic infection^24^. More recently, we showed that perinatal exposure to BPA via oral gavage of mothers induced intestinal and systemic immune imbalances in young adult male and female offspring mice, through the modulation of splenic and intestinal Th1/Th17 immune responses^25, 26^. These studies highlighted that perinatal BPA exposure via mothers can interfere with the maturing immune system, providing information that warrants major consideration for human safety^27^. Due to compiling evidence demonstrating that BPA exposure is associated to immune related diseases (allergy, inflammatory bowel diseases, food intolerance)^28, 29^, and due to the similarities in BPA’s analogue structures (BPS and BPF), the question raised whether those substitutes are safe or not via dermal exposure of mothers. Indeed, in adults and according to the EFSA, mean BPA exposure through dermal route represents nearly the tierce of all exposure routes (oral exposure being the main route almost 2 tierces)^30^. Yet, no study has investigated the immunotoxicity of BPS and BPF after perinatal exposure through dermal route in vivo. In this context, the objective of the present study is to compare the effect of mothers’ dermal exposure during gestation and lactation to two doses of BPA, BPS or BPF (5 and 50 µg/kg of BW/d) on immune system at intestinal and systemic levels of male and female offspring mice.

## Results

### Birth rate and body weight of offspring mice

**Figure 1:**
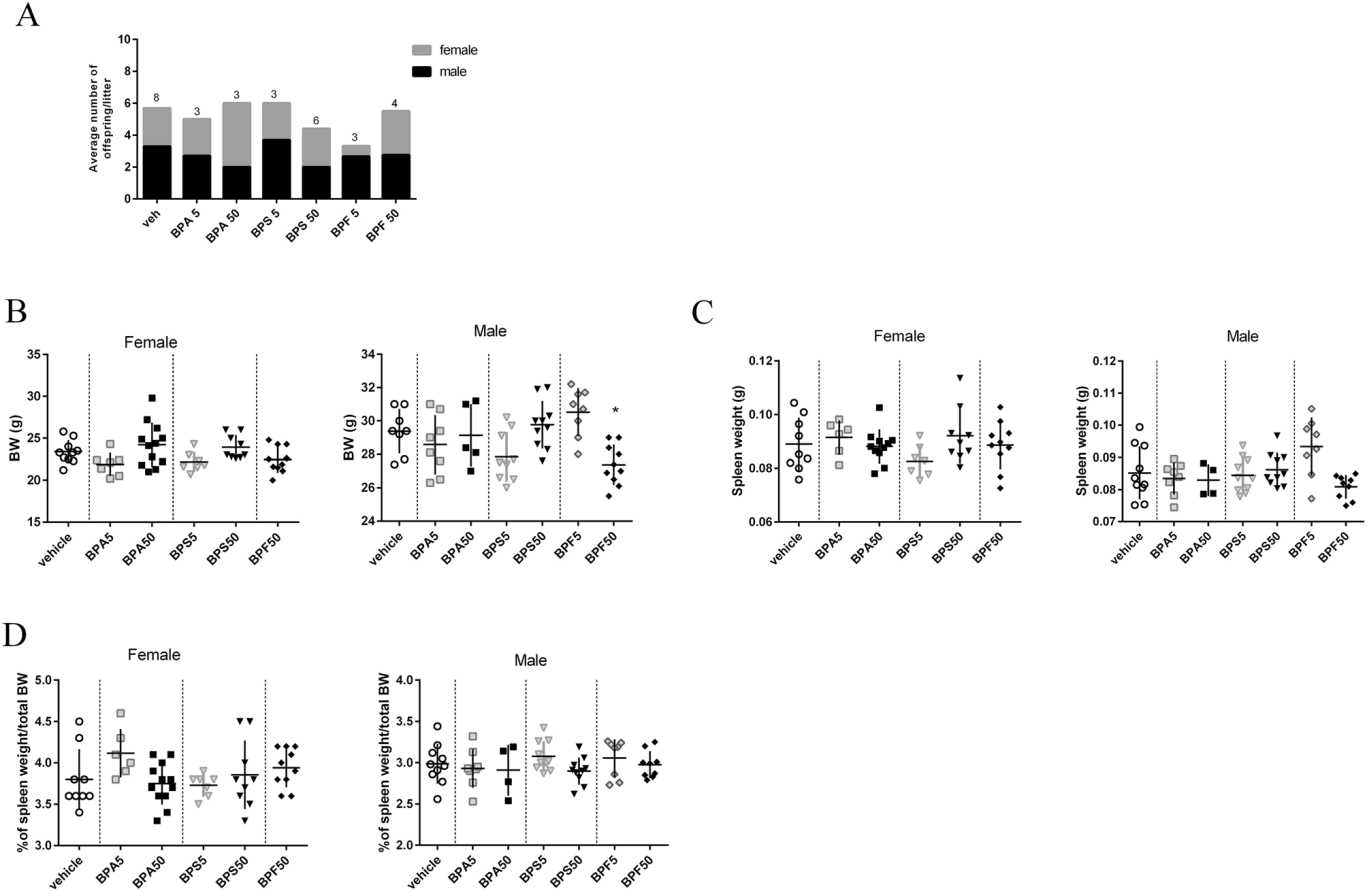
Consequence of dermal exposure to bisphenols on birth rate and BW of adult offspring mice. (**A**) Number of birth and sex of the offspring in vehicle, BPA, BPS and BPF groups (male: black box; female: grey box). The number at the top of each column correspond to the number of mothers used for each treatment group. (**B**) Body weight of female and male mouse offspring in the different treatment groups at PND70. (**C**) Weight of spleen in female and male offspring at PND70. (**D**) Percent of spleen weight/total BW of female and male offspring determined in the different treatment groups at PND70. *P < 0.05; **P < 0.01 vs. vehicle group. N = 5–12 offspring mice per group.

Whereas the sex-ratio was quite the same in all groups (Fig. 1A), the BPF5 group was composed by 8 males and only 2 females (from 3 different mothers), causing the withdrawal of BPF5 female offspring group in our study (Supplementary data Fig. 1). Neither BPA nor BPS perinatal exposure through dermal route changed the body weight (BW) or the spleen weight of male and female offspring (Fig. 1B–D). However, perinatal exposure to BPF at 50 μg/kg BW/d led to a significant decrease of the body weight of male offspring (Fig. 1B).

### Intestinal immune response of offspring mice

**Figure 2:**
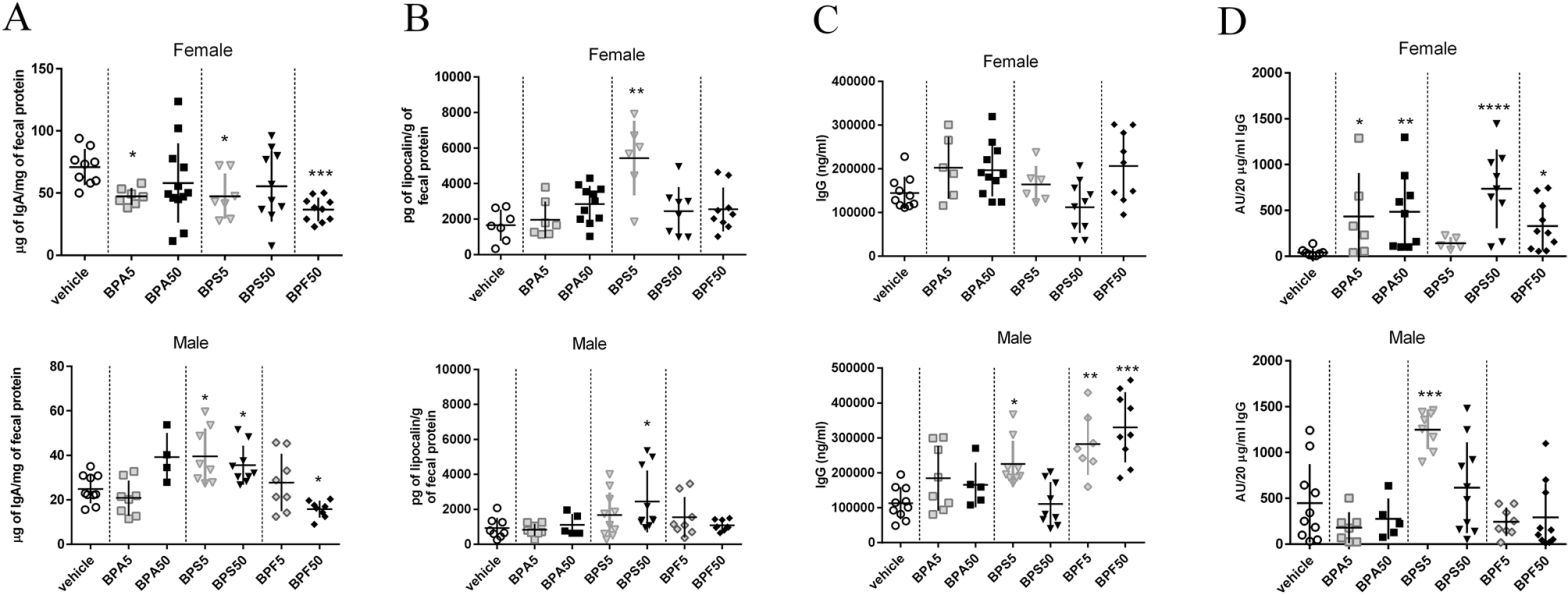
Impact of dermal exposure to bisphenols on intestinal IgA production, intestinal inflammation, total IgG and specific IgG responses against *E. coli* in adult offspring mice. (**A**) Total IgA concentration measured by ELISA in fecal samples of female and male offspring mice at PND70. (**B**) Lipocalin concentration determined in fecal supernatant of female and male offspring mice at PND70. (**C**) Plasma IgG concentration measured by ELISA. (**D**) IgG specificity against *E. coli* assessed by ELISA after normalizing to IgG concentrations. *P < 0.05; **P < 0.01; ***P < 0.001 vs. vehicle group. N = 5–12 offspring mice per group.

Interestingly, we observed a reduction of fecal IgA level after BPA5, BPS5 and BPF50 exposure (Fig. 2A), and an increase of lipocalin concentration in BPS5 female offspring group (Fig. 2B). In contrary, fecal IgA concentration of male offspring was significantly increased in BPS groups, and decreased in BPF50 group (Fig. 2A). We noticed a significant increase of fecal lipocalin concentration only in BPS50 male offspring group (Fig. 2B). In females, plasmatic total IgG concentrations were similar for all the groups compared to controls (Fig. 2C). However, specific anti-*E. coli* IgG concentration was significantly increased in plasma of BPA groups (5 and 50 μg/kg BW/d), and in BPS50 and BPF50 groups in female offspring (Fig. 2D).

We also noticed a significant increase of plasmatic IgG in male offspring in BPS5, BPF5, and BPF50 groups (Fig. 2C), but only BPS5 group had a significant increase of specific anti-*E. coli* IgG in plasma compared to control group (Fig. 2D). Then, immune response is differently affected by BPs in male and female.

### Intestinal and systemic cellular immune responses

**Figure 3:**
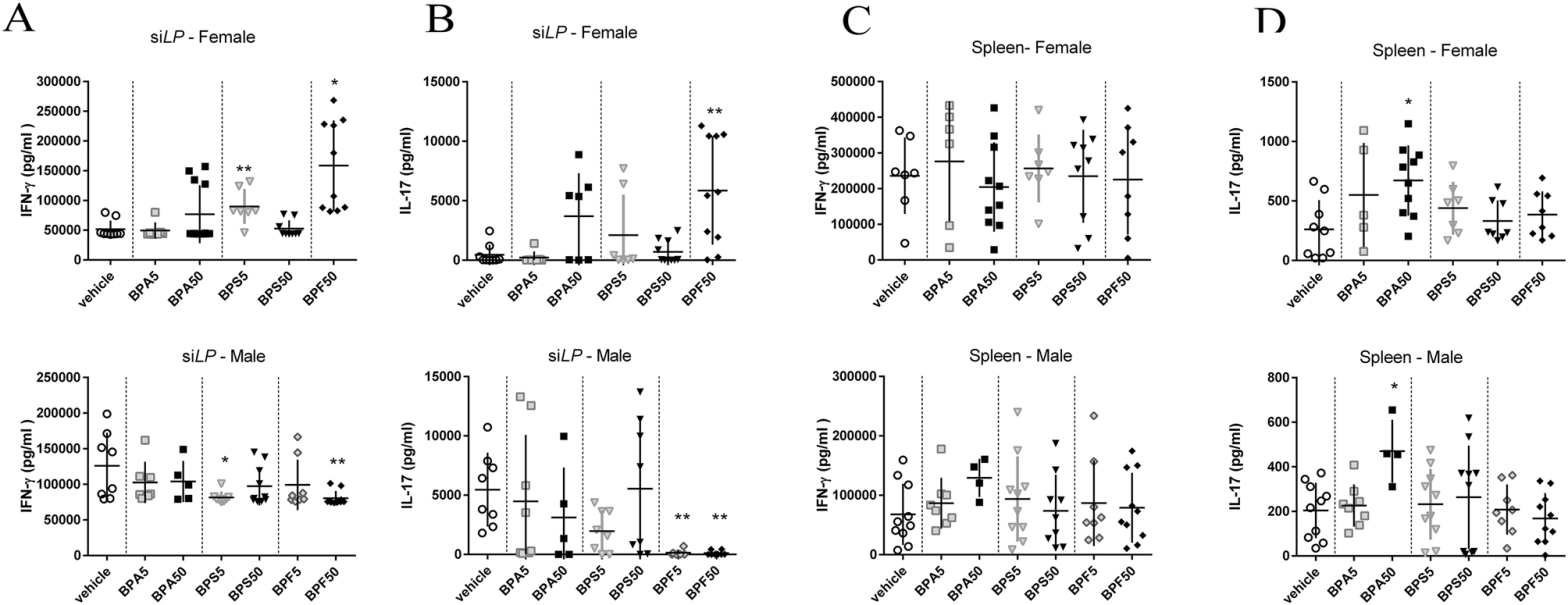
Effects of dermal exposure to bisphenols on Th1/Th17 immune response in adult offspring mice. IFN-γ (**A**,**C**) or IL-17 (**B**,**D**) secretion after anti-CD3/CD28 in vitro restimulation of isolated cells from si*LP* (**A**,**B**) or spleen (**C**,**D**) of female and male offspring mice at PND70. *P < 0.05; **P < 0.01 vs. vehicle group. N = 5–12 offspring mice per group.

In female offspring, we observed an increase of Th1 subpopulation in small intestine *lamina propria* (si*LP*) of BPA50 group (Supplementary data Fig. 2A) without increasing IFN-γ secretion in response to TCR stimulation (anti-CD3/CD28) (Fig. 3A). IFN-γ secretion in supernatant of si*LP* was increased after BPS5 and BPF50 exposure in female offspring mice and decreased after BPS5 and BPF50 exposure in male offspring mice compared to control group (Fig. 3A). A significant increase of Th17 frequency was noticed for BPA50 female offspring (Supplementary data Fig. 2B) associated with a non-significant increasing trend in IL-17 secretion in supernatant of si*LP* culture (after anti CD3/CD28 stimulation) (Fig. 3B). We also observed an increased IL-17 secretion in BPF50 female offspring offspring which was not associated with an increase of Th17 frequency (Supplementary data Fig. 2B). In contrast, in male offspring mice, IL-17 secretion of si*LP* cells was significantly reduced in BPF5 and BPF50 groups compared to control group (Fig. 3B). Cytokine levels are differently affected by BPs in male and female at intestinal level.

No significant change in IFN-γ secretion was observed after anti-CD3/CD28 stimulation in male and female offspring mice (Fig. 3C and Supplementary data Fig. 2C) nor in Th1 frequency in female offspring mice. We also analyzed the Th17 frequency at systemic level. A significant decrease of Th17 frequency was noticed in female offspring mice (Supplementary data Fig. 2D). Moreover, a significant increase of IL-17 secretion was noticed in splenocyte supernatant (after anti-CD3/CD28) stimulation of BPA50 group in both sexes (Fig. 3D).

### Activated and regulatory T cell frequency

**Figure 4:**
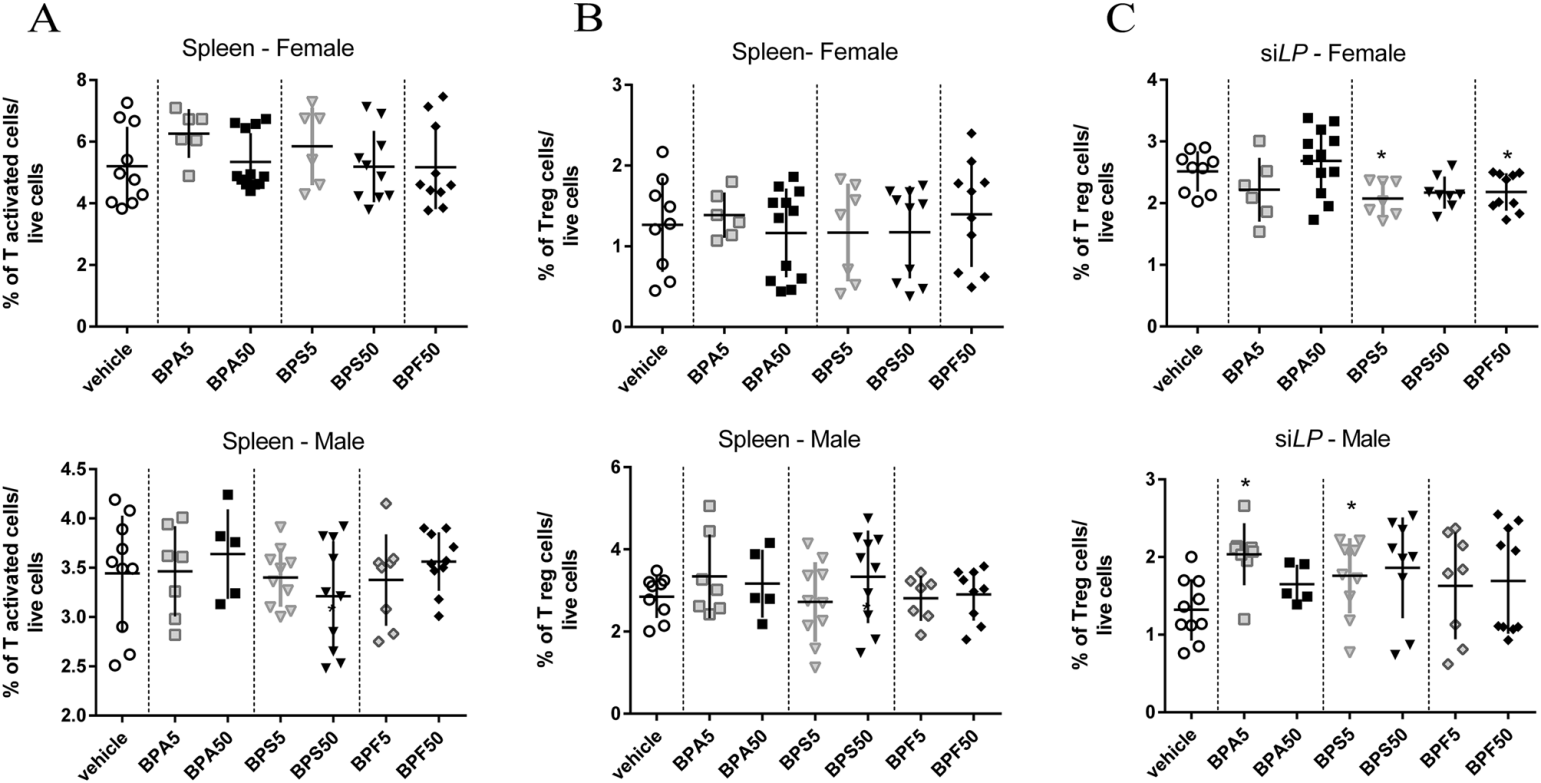
Effects of dermal exposure to bisphenols on the immune homeostasis of adult offspring mice. Proportion of CD4^+^CD44^high^CD62L^low^ T-activated cells in spleen (**A**) and CD4^+^CD25^+^FoxP3^+^ Treg cells in spleen (**B**) and si*LP* (**C**) of female and male offspring mice at PND70. *P < 0.05 vs. vehicle group. N = 5–12 offspring mice per group.

Mother exposure to the different BPs via dermal route during gestation and lactation did not induce any changes in activated and regulatory T cell frequency at systemic level in male or female offspring mice (Fig. 4A,B).

At intestinal level, we observed a significant decrease of Treg cells in si*LP* of BPS5 and BPF50 female offspring (Fig. 4C). In opposite, in male offspring, Treg frequency was increased in si*LP* of BPA5 and BPS5 groups compared to control group (Fig. 4C). Immune cell populations are differently affected by BPs in male and female.

### Colonic and jejunal IFN-γ and IL-17 concentrations in offspring mice

**Figure 5:**
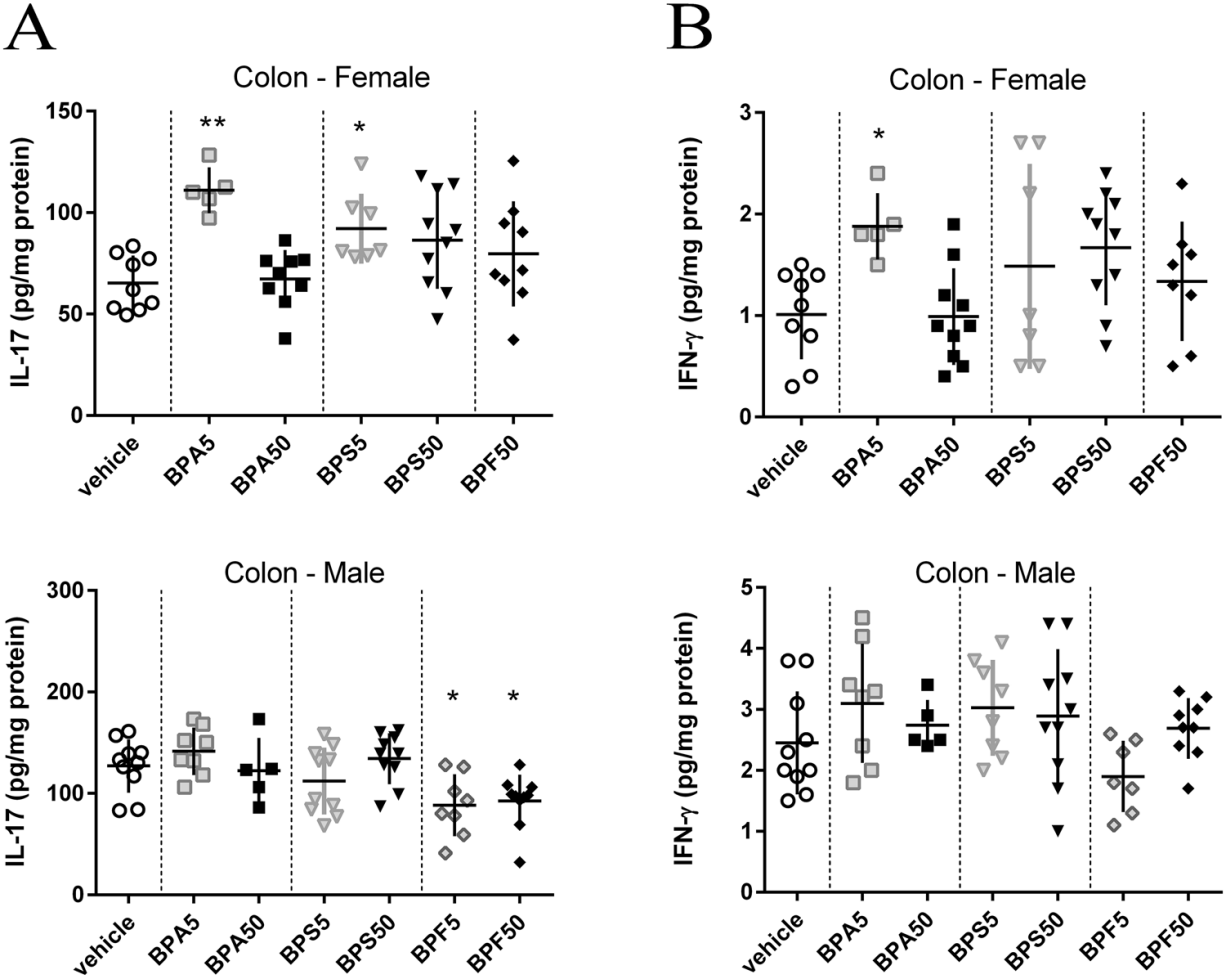
Impact of dermal exposure to BPA on potent IL-17 and IFN-γ inflammation in colon of adult offspring mice. IL-17 (**A**) and IFN-γ (**B**) levels in colonic samples from female and male offspring mice at PND70. *P < 0.05; **P < 0.01 vs. vehicle group. N = 5–12 offspring mice per group.

As shown in Fig. 5A,B, a significant increase of colonic IL-17 concentration was observed in female offspring after BPA5 and BPS5 treatment compared to control group, whereas a significant increase of IFN-γ was only observed for BPA5. In male offspring mice, a decrease of IL-17 concentration was observed in colon at adulthood after BPF5 and BPF50 treatment without change in IFN-γ concentration. Cytokines concentrations in colon are differently affected by BPs in male and female. In contrast, at jejunal level, a significant increase of IFN-γ concentration in female groups was observed after mother’s exposure to BPA5 and BPF50, whereas a decrease of IL-17 level was noticed in BPS5 female offspring group (Supplementary data Fig. 3A,B). No difference was reported in male offspring mice.

### Sex-dimorphism and discriminative parameters revealed through multivariate analyses

**Figure 6:**
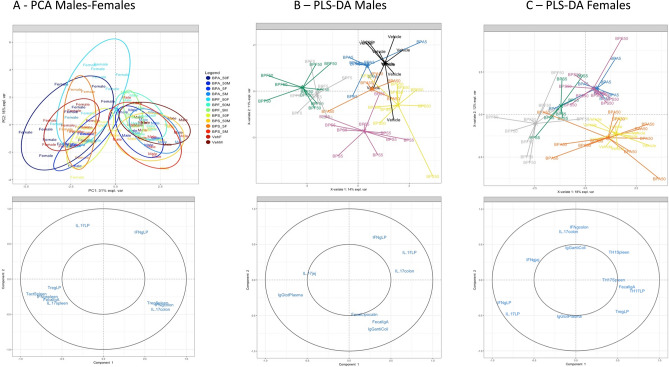
Multivariate analysis representing immune profiles in function of BP dermal exposure and sex in adult offspring mice. (**A**) Sample score plot and associated loading plot on the first two PCA components resulting from all data set (without BW associated parameters) in males and females offspring mice. Each color indicated groups for each sex with 0.85% confidence level ellipse plots. (**B**) PLS-DA sample score plot and associated loading plot on the first two components derived from data set from all treated groups in male offspring mice (without BW associated parameters). (**C**) PLS-DA score plot and associated loading plot on the first two components derived from data set from all treated groups in female offspring mice (without BW associated parameters). Only loadings with correlation threshold > 0.5 were represented on the loading plots. % expl var: percentages for each first two components explained by the model*.* N = 5–12 offspring mice per group.

Based on the compilation of all the humoral and cellular immune associated data sets, a non-supervised method (Principal Component Analysis, PCA) was first performed to explore diversity patterns of responses according to sex, BP dermal exposure and doses. Sample plot revealed a clear separation of mice according to their sex along the first component regardless of the BPs or doses used, demonstrating an important dimorphism effect in our study (Fig. 6A). The loading plot associated to the PCA demonstrated that the increased inflammatory parameters in colon and IFN-γ in si*LP* was more pronounced in males, whereas females were characterized by higher systemic response and higher level of fecal IgA and IL-17 in si*LP*. Interestingly, if we focus only on data set from the vehicle group, we obtained also a strong separation of mice according to sex, demonstrating the importance of focusing separately on each sex when studying host responses such as systemic and intestinal immune response (Supplementary data Fig. 4).

The next step was to perform a supervised analysis (PLS-DA) on male offspring data set, in order to identify a host-response signature (related to intestinal and systemic immune responses characterizing each sort and dose of BP (Fig. 6B). The model used allowed us to discriminate the vehicle group (black line) from treated BP groups, BPF treated mice and BPS being the most distant ones. BPS and BPF show indeed a stronger separation from the control group even at low dose of 5 μg/kg BW/d (Fig. 6B). The loading plot showed that concentrations of IL-17 according to the localization (colon, jejunum or si*LP*) were important contributors to the discrimination between control and BPF treated male mice. Likewise, fecal lipocalin and IgA associated to anti*-E. coli* IgG levels contributed mainly to differentiate BPS treated male mice from controls.

In female offspring, aside from mice treated with BPA at high dose, all BP treated mice clustered separately from the vehicle group (Fig. 6C). Among the analyzed immune parameters, both IL-17 and IFN-γ concentrations according to localization (si*LP* or colon) were key cytokines able to separate group of mice according to their BPs dermal exposure.

## Discussion

The immune response is primed in utero by mother microbial metabolites, and differs from males and females. In fact, sex is an important variable that influences both innate and adaptive immune system development^31^. Thereby, dysregulations during the “window of opportunities” might favor development of diseases in later life in a sex-specific manner^32^. Indeed, the perinatal period is a critical window for BPA exposure, which has sex-dependent impact on immune system development and function in life^24, 26^. However, the role of different bisphenols in the occurrence of inflammation remain unclear^25, 33^.

Based on these findings and questions, the current study focused on dysregulations in development of both intestinal and systemic immune homeostasis of male and female offspring mice, provoked by perinatal exposure to BPA, BPS and BPF after mother dermal impregnation.

In our study, the lowest birth rate was observed when mothers were treated with BPF5 through dermal exposure in comparison to control mothers. It is well known that BPA is characterized by a pronounced influence on hormonal regulation. A recent review of in vitro, in vivo and epidemiologic studies showed associations between BPA levels and fetal implantation failure in women, birth rate, birth body weight and length of gestation^34, 35^ revealed also that these chemicals have a direct negative effect on maternal, fetal, and neonatal outcomes, including rates of preterm birth with developmental defects and birthweight. Wan et al. (2018) evaluated the impact of maternal BPS exposure on birth outcomes in human studies. In this Chinese cohort, BPS exposure of mother did not affect birth weight or length but high concentration of maternal urinary BPS was associated with increased gestational duration^36^. Few data are available on the effects of BPF on birth outcome. However, a study showed that BPF also showed other effects such as altered reproductive organ weights and reproductive end-points, that could explain the low birth rate we observed in the BPF group^37^.

Our results also demonstrated that exposure to the different BPs, even at low doses, induced a decrease of fecal IgA in offspring female adults and, on the contrary an increase in male offspring highlighted sex-dependent effects. Intestinal IgA is involved in the development and maintenance of the homeostasis between the host immune system and commensal microbiota^38^. The fall of IgA in female offspring was associated with an increased concentration of lipocalin, an inflammation marker, in feces after BPS exposure in female. These results are in accordance with our previous studies showing a reduced IgA production after oral perinatal exposure of BPA^26^. An aberrant commensal microbiota implantation can lead to a reduced IgA production by the intestine, which could explain our results^39^. Indeed, we also observed an increase of anti-*E. coli* IgG in plasma of female offspring after mother’s impregnation with BPA at 5 or 50 µg/kg BW/d, and with BPS or BPF at 50 µg/kg BW/d. This was consistent with results obtained after oral administration of BPA in female offspring mice^26^, revealing the potential impacts of BPs’ dermal exposure on gut microbiota homeostasis. Interestingly, BPS impregnation at low dose provoked a significant increase of anti-*E. coli* IgG in male offspring associated with high lipocalin concentration in feces, adding impaired intestinal immune response evidence in male offspring too. We also observed differences in cytokines expression in gut after BP perinatal dermal exposure. Those cytokines signatures after perinatal BPs exposure are different in male and female offspring. A significant increase of IL-17 and IFN-γ concentrations was measured in colons of female offspring mice after not only dams’ dermal exposure to BPA5 but also to BPS5 (for IL-17), reflecting colonic inflammation. A significant rise of IFN-γ cytokine was observed in the jejunum after BPF dams’ dermal exposure, without affecting colon, suggesting a specific mechanism of action of this contaminant. Indeed, a recent study showed that BPF, contrary to BPA, can induce immunotoxicity in mouse macrophages at environmentally relevant concentrations^40^.

In the gut, inflammatory Th17/Th1 and regulatory T cells (Treg) contribute to the immune system homeostasis. Th17 cells is the most abundant CD4^+^ T cells in mucosal tissues^41^. They secrete isoforms of IL-17 and/or IL-22, which confer protection against fungi and pathogenic bacteria. In the present study, BPA and BPF at 50 μg/kg BW/d provoked a strong increase of Th1 and Th17 cell-mediated inflammation after in vitro anti-CD3/CD28 restimulation of intestinal immune cells from female offspring mice. These results are in accordance with our previous work revealing that perinatal exposure to BPA after oral administration induces a potent Th1/Th17 signature at local level^25^. Opposite, in male offspring, we observed a decrease of IL-17 (BPS at 5 μg/kg BW/d and BPF at 50 μg/kg BW/d) and IFN-γ (BPF at both doses) production by *lamina propria* anti-CD3/CD28 restimulated cells. Similar results suggesting sex-dependent response were observed when mice were orally exposed to BPA during the perinatal period^25^. Luo et al. (2016) described also a sex-specific persistent increase of Th17 after perinatal exposure to BPA, with more pronounced changes in female than in male offspring mice^42^.

At the systemic level, we also noticed a higher IL-17 level after anti-CD3/CD28 restimulation of splenocytes, but only in BPA50-exposed female offspring after perinatal dermal exposure. Luo et al. (2016) reported similar observation after gestational and lactational exposure to BPA^42^. Recent data published by Gayrard et al. (2019) indicated that only 41% of BPS was glucuronidated by the liver after oral absorption producing a systemic bioavailability of 57.4%^19^. Then, the oral BPS systemic exposure was on average about 250 times higher than for BPA for an equal oral molar dose of the two compounds due to the higher systemic availability of BPS and its 3.5 times lower plasma clearance. However, our results show greater systemic effects after dermal exposure to BPA compared to BPS. Then, the cutaneous versus oral route of exposure involves different mechanisms of bioavailability.

It is well known that Treg cells participate to immune tolerance^43^. We observed, here, a decrease in Tregs frequency from siLP in female offspring exposed to BPS and BPF at 5 and 50 μg/kg BW/d, respectively. These results are in accordance with those obtained by Malaisé et al. (2018) in female offspring mice after BPA perinatal exposure by oral route. In contrast, in male offspring mice, we noticed an increase of Treg subsets after dermal exposure of mothers to low dose of BPA and BPS, which was similar to what we reported for mice orally exposed to BPA during the perinatal period^25^.

Several studies support that exposure to low-dose of BPA leads to sexual dimorphism^44, 45^, but no one described this phenomenon for BPS and BPF. We observed a clear separation between our control males and females with PCA and PLS-DA analysis that could be explained by already described sex-dependent basal differences in immune cell frequencies and responses^31^. In BP treated groups, sex-specific effects that we observed with PCA and PLS-DA analysis are consistent with previous studies revealing that perinatal exposure to BPA can provoke inflammation, hormone level modulations as well as behavior changes^46^ in a sex-dependent manner.

In the present study, dermal exposure to BPA and BPF during gestational and lactational period caused more significant changes in female offspring mice in terms of cellular immune responses inducing an intestinal Th1/Th17 inflammation. Our findings suggest that perinatal exposition to an environmentally relevant dose of BPA and BPF results in changes in Th1 and Th17 development, which may contribute to their developmental immunotoxicity. Moreover, IL-17-secreting Th17 cells are key players to promote inflammatory diseases in mice^47, 48^. Indeed, strong evidence revealed that Th17 cells represent a distinct subset of CD4^+^ T lymphocytes that play a critical role in chronic inflammation and autoimmunity in mice^49^. While the pro-inflammatory properties of IL-17 are responsible for host-protective ability, unrestrained IL-17 signaling is associated with immunopathology, autoimmune disease and cancer progression. An uncontrolled acceleration of the system or failure of the brakes can both lead to persistent inflammation resulting in tissue damage and NCDs development later on. Biologically, it was imperative that the precarious balance between pathogenic and protective sides of IL-17 signaling must be maintained^49^. In our previous study, we showed that weaker Th17/Th1 responses provoked by BPA in the small intestine is associated with intestinal dysbiosis at adulthood, leading to metabolic disorders such as type 2 diabetes and obese phenotype in aged offspring male mice^25^. Interestingly, our present study revealed similar impacts in immune responses after dermal exposure and sex-dependent effects. Here, we showed that BPs dermal exposure of mother could impair differentially immune response of offspring in gut and at systemic level. Those data obtained at steady state question the safety of BPs and their identification as risk factor for development of immunological pathologies. Yet, these experimental findings request further experimental and epidemiological studies to assess the effects of BPs on the risk of developing immune-mediated diseases during childhood and in adults after perinatal exposure.

We demonstrated that BPA substitutes, BPS and BPF, are also able to affect intestinal and systemic immune systems of offspring after dermal exposure of mothers, questioning their iniquitous use to replace BPA. Those results pave the way for further studies regarding the consequences of perinatal dermal exposure to BPS and BPF. Moreover, we also demonstrate that dermal exposure of mother to BPs impairs the immune response of offspring in a dose and sex dependent manner.

In conclusion, this work provides structural data on the consequences of BPs dermal exposure of mothers on offspring immune response at steady state. This explorative study will give essential knowledge that will condition further studies regarding the consequences of BPs dermal exposure of mothers on immune related pathologies, and highlights the urgency of further studies on the development of inflammatory immune diseases considering the sex-specific effects of each different BPA substitutes.

## Materials and methods

### Animals and BP treatments

The study was carried out in compliance with the ARRIVE guidelines (http://nc3rs.org.uk/arrive-guidelines). All experimental protocols were conducted in compliance with French legislation (Decree: 2001-464 29/05/01) and EEC regulations (86/609/CEE) governing the care and use of laboratory animals, after validation by the local ethics committee for animal experimentation (Comité d’Ethique pour l’Expérimentation Animale Toxalim Toulouse, TOXCOM/0151/LG, APAFiS #4583).

We used C3H/HeN (Janvier, Roubaix, France) mice known to be excellent breeders to minimize stress induced by the treatment during perinatal period. Perinatal experiment was conducted as previously described^25^, excepted that pregnant and lactating mice were daily exposed by dermal route (instead of oral route) from gestation day 15 to weaning of pups (day21; d21). To this aim, 10 μl of BPA, BPS or BPF at 5 or 50 µg/kg of BW diluted in ethanol 70% were daily dropped off on the neck of the mothers. The 10 μl drop off on the neck does not allow the mouse to lick it, avoiding oral contamination, and the absence of shaving prevent an unappropriated leak of BP in blood circulation.

We chose to use ethanol 70% as a vehicle of BP as Biedermann et al. (2010) previously investigated the role of ethanol, found in hand creams, as a vector for skin penetration^50^. BPA applied to the finger pad with ethanol rapidly entered the skin to such an extent that after about 1 h it was no longer extractable even with ethanol for 30 s. They did show that BPA can enter the skin to a depth such that it is no longer removable by washing hands^50^. These findings showed action of ethanol as a transfer vector allowing skin penetration and dermal exposure.

It is known that bisphenol dermal exposure is very specific to thermal paper manipulation and/or cosmetics. However, thermal papers contain exclusively free bisphenols, meaning that people, and more precisely cashiers, are handling free bisphenols with their hands. In fact, it has been shown that using hand sanitizer two seconds after handling thermal paper released 235 μg of BPA on hands^51^.

We also chose to work with 5 and 50 µg/kg BW/d based on data from literature. Indeed, Mielke et al. (2011) simulated the concentration of BPA in blood after dermal transfer from thermal paper by using physiologically based toxicokinetic modeling^50^. Their results showed comparable range of BPA concentrations between dermal and oral exposure. Indeed, the estimated daily intake (EDI, occupational exposure) via dermal transfer from cash receipt has been calculated in the range of 18–3119 ng/kg BW/d^3^. These data confirm the relevance of the two doses (5 and 50 µg/kg BW/d). For more clarity, these groups will be referred as BPA5, BPA50, BPS5, BPS50, BPF5 and BPF50. The vehicle alone (ethanol 70%) was used as control group. All animals were kept at a constant temperature (22 ± 1 °C) and maintained on a 12:12 h light/dark cycle (light on at 7:30 am). Females and males perinatally exposed through their dams were then used as experimental mice, except for BPF5 group where only male offspring were used, due to low female births (Fig. 1). The experiment was conducted on more than three litters/treatment and at least three animals born of each different litter were used for each measurement in order to minimize potential litter effect. Furthermore, litters were normalized in number n = 5 ± 1 and in sex-ratio^52^ and at weaning, pups within the same treatment but from different litters were mixed. At PND70, offspring were euthanized, and blood, jejunum, colon and feces were harvested. Small intestine *Lamina Propria* (si*LP*) and spleen were collected for primary cell culture. Sample size ranged from n = 5 to 12 for analyses and the exact number in each group is mentioned in Supplementary data Fig. 1.

### Humoral response in plasma and feces

Intracardiac blood was collected with a heparinized syringe, and plasma were kept at − 80 °C. Fecal proteins were isolated through mechanical extraction in PBS with complete antiprotease cocktail (Roche Diagnostic, Meylan, France) and frozen at − 80 °C. Plasma and fecal IgG and IgA concentrations were measured by ELISA as described previously^26^.

### Immunoglobulin specificity against commensal *E. coli* lysate

Anti- *E. coli* IgG lysate was measured as described previously^53^. Results were expressed in comparison with a standardized immune serum as arbitrary units (AU) per 20 μg/mL of IgG.

### Spleen and si*LP* cell isolation

Spleens were dissociated with PBS-1% KnockOutTM SR (KO SR) medium (Gibco) into splenic single-cell suspensions by using a 70 μm cell strainer. Small intestines were first incubated in cold PBS, cut into 0.5 cm pieces, washed four times in 30 mL of PBS with 3 mM EDTA (Sigma-Aldrich) and digested in 20 mL of DMEM added with 20% FCS and 100 U/mL of collagenase (Sigma-Aldrich) for 40 min at 37 °C. Si*LP* cells were purified on a 40–80% Percoll gradient run for 15 min at 1800*g* at RT. The same techniques detailed below were used to assessed cell population and their functionality in intestine and at systemic levels.

### Flow cytometry analysis

Splenic cells and isolated cells from si*LP* were stained with specific antibodies against mouse as follows: activated T-cells: CD4 (BD), CD44 (BD), CD62L (BD); regulatory T-cells: CD4 (BD), CD25 (BD), Foxp3 (ebioscience); Th17: CD3 (BD), RORγt (BD), IL-17 (BD) and Th1: CD3 (BD), T-bet (BD) and IFN-γ (BD). Surface and intracellular staining were performed as previously described^26^.

### Cytokine measurements

Si*LP* and splenic cells were also seeded on 24-well plates at 1 × 10^6^ cells per well for cytokines assays in Cerrotini culture medium (Malaisé et al., 2018) in presence or absence of 5 μg/mL hamster anti-mouse CD3 and hamster anti-mouse CD28 (BD biosciences) coated wells. Culture supernatants were collected after 3 days of stimulation and frozen at − 80 °C prior to cytokines secretion measurement. Cytokines were also analyzed in supernatant of jejunal, colonic fragments or feces resuspended in RIPA buffer as previously described^26^. Jejunal, colonic or fecal protein concentrations were measured using BCA uptima kit (Interchim).

IL-17, IFN-γ and lipocalin were assayed using commercial ELISA kits (R&D Systems, Lille, France), following manufacturer’s instructions.

### Multivariate data processing

Mixomics package (6.8.2 version) with RStudio software (Boston, MA) (https://rstudio.com, 1.0.44 version) was used to build, first a principal component analysis based on the compilation of all data obtained in study. In a second, two Partial Least-Squares Discriminant Analysis (PLS-DA) were built separately for each sex to depict immune signature associated with sex and BPs treatment. PLS-DA is a multivariate supervised approach that operates by projecting the samples (X) onto a low-dimensional space of so-called latent variables that maximizes the separation between different groups of samples according to their class labels (Y = mice treatments). Repeated Mfold cross-validations were used to select the optimal number of latent variables for PLS-DA models with minimal error rate.

### Statistical analysis

Statistical analysis was performed using GraphPad Prism version 6.00 (GraphPad Software, San Diego, California, USA, https://www.graphpad.com/scientific-software/prism). Results were expressed as means ± SEM. Kruskal–Wallis one-way ANOVA comparing each BP group (5 and 50 µg/kg BW/d) to the vehicle group followed by Dunn’s post hoc for multiple comparisons were used for statistical analysis. P-values < 0.05 were considered significant (indicated by asterisks): *P < 0.05; **P < 0.01; ***P < 0.001; ****P < 0.0001.

## Supplementary Information

Supplementary Figure 1.

Supplementary Figure 2.

Supplementary Figure 3.

Supplementary Figure 4.

Supplementary Caption.

## References

[1] EFSA Panel on Food Contact Materials, Enzymes, Flavourings and Processing Aids (CEF) (2015). Scientific opinion on the risks to public health related to the presence of bisphenol A (BPA) in foodstuffs: Opinion on BPA. EFSA J..

[2] von Goetz N (2017). Including non-dietary sources into an exposure assessment of the European Food Safety Authority: The challenge of multi-sector chemicals such as Bisphenol A. Regul. Toxicol. Pharmacol..

[3] Björnsdotter MK, de Boer J, Ballesteros-Gómez A (2017). Bisphenol A and replacements in thermal paper: A review. Chemosphere.

[4] Goldinger DM (2015). Endocrine activity of alternatives to BPA found in thermal paper in Switzerland. Regul. Toxicol. Pharmacol..

[5] Sogorb MA, Estévez J, Vilanova E (2019). Case study: Is bisphenol S safer than bisphenol A in thermal papers?. Arch. Toxicol..

[6] Lotti N, Colonna M, Fiorini M, Finelli L, Berti C (2011). Poly(butylene terephthalate) modified with ethoxylated bisphenol S with increased glass transition temperature and improved thermal stability. Polymer.

[7] Liao C (2012). Occurrence of eight bisphenol analogues in indoor dust from the United States and several Asian countries: Implications for human exposure. Environ. Sci. Technol..

[8] Wu L-H (2018). Occurrence of bisphenol S in the environment and implications for human exposure: A short review. Sci. Total Environ..

[9] Ye X (2015). Urinary concentrations of bisphenol A and three other bisphenols in convenience samples of U.S. adults during 2000–2014. Environ. Sci. Technol..

[10] Danzl E, Sei K, Soda S, Ike M, Fujita M (2009). Biodegradation of bisphenol A, bisphenol F and bisphenol S in seawater. Int. J. Environ. Res. Public Health.

[11] Molina-Molina J-M (2013). In vitro study on the agonistic and antagonistic activities of bisphenol-S and other bisphenol-A congeners and derivatives via nuclear receptors. Toxicol. Appl. Pharmacol..

[12] Kojima H (2019). Profiling of bisphenol A and eight of its analogues on transcriptional activity via human nuclear receptors. Toxicology.

[13] Viñas R, Watson CS (2013). Bisphenol S disrupts estradiol-induced nongenomic signaling in a rat pituitary cell line: Effects on cell functions. Environ. Health Perspect..

[14] Fiege, H. *et al.* Phenol Derivatives. in *Ullmann’s Encyclopedia of Industrial Chemistry* (ed. Wiley-VCH Verlag GmbH & Co. KGaA) (Wiley-VCH Verlag GmbH & Co. KGaA, 2000). 10.1002/14356007.a19_313.

[15] Fic A, Žegura B, Sollner Dolenc M, Filipič M, Peterlin Mašič L (2013). Mutagenicity and DNA damage of bisphenol A and its structural analogues in HepG2 cells. Arh Hig Rada Toksikol..

[16] Ruan T (2015). Evaluation of the in vitro estrogenicity of emerging bisphenol analogs and their respective estrogenic contributions in municipal sewage sludge in China. Chemosphere.

[17] Cabaton NJ (2013). Effects of low doses of bisphenol A on the metabolome of perinatally exposed CD-1 mice. Environ. Health Perspect..

[18] Corbel T (2014). Bidirectional placental transfer of Bisphenol A and its main metabolite, Bisphenol A-Glucuronide, in the isolated perfused human placenta. Reprod. Toxicol..

[19] Gayrard V (2019). Oral systemic bioavailability of bisphenol A and bisphenol S in pigs. Environ. Health Perspect..

[20] Bansal A (2017). Sex- and dose-specific effects of maternal bisphenol A exposure on pancreatic islets of first- and second-generation adult mice offspring. Environ. Health Perspect..

[21] Mikołajewska K, Stragierowicz J, Gromadzińska J (2015). Bisphenol A—Application, sources of exposure and potential risks in infants, children and pregnant women. Int. J. Occup. Med. Environ. Health.

[22] Spanier AJ (2012). Prenatal exposure to bisphenol A and child wheeze from birth to 3 years of age. Environ. Health Perspect..

[23] Trasande L, Attina TM, Blustein J (2012). Association between urinary bisphenol A concentration and obesity prevalence in children and adolescents. JAMA.

[24] Menard S (2014). Food intolerance at adulthood after perinatal exposure to the endocrine disruptor bisphenol A. FASEB J..

[25] Malaisé Y (2017). Gut dysbiosis and impairment of immune system homeostasis in perinatally-exposed mice to Bisphenol A precede obese phenotype development. Sci. Rep..

[26] Malaisé Y (2018). Consequences of bisphenol A perinatal exposure on immune responses and gut barrier function in mice. Arch. Toxicol..

[27] Hessel EV (2016). Assessment of recent developmental immunotoxicity studies with bisphenol A in the context of the 2015 EFSA t-TDI. Reprod. Toxicol..

[28] Xu J, Huang G, Guo T (2016). Developmental bisphenol A exposure modulates immune-related diseases. Toxics.

[29] Xu J, Huang G, Nagy T, Guo TL (2019). Bisphenol A alteration of type 1 diabetes in non-obese diabetic (NOD) female mice is dependent on window of exposure. Arch. Toxicol..

[30] Ćwiek-Ludwicka K (2015). Bisphenol A (BPA) in food contact materials—New scientific opinion from EFSA regarding public health risk. Rocz Panstw Zakl Hig.

[31] Klein SL, Flanagan KL (2016). Sex differences in immune responses. Nat. Rev. Immunol..

[32] Ellsworth L, Harman E, Padmanabhan V, Gregg B (2018). Lactational programming of glucose homeostasis: A window of opportunity. Reproduction.

[33] van Esterik JCJ (2014). Programming of metabolic effects in C57BL/6JxFVB mice by exposure to bisphenol A during gestation and lactation. Toxicology.

[34] Pergialiotis V (2018). Bisphenol A and adverse pregnancy outcomes: A systematic review of the literature. J. Matern. Fetal Neonatal Med..

[35] Tomza-Marciniak A, Stępkowska P, Kuba J, Pilarczyk B (2018). Effect of bisphenol A on reproductive processes: A review of in vitro, in vivo and epidemiological studies. J. Appl. Toxicol..

[36] Wan Y (2018). Relationship between maternal exposure to bisphenol S and pregnancy duration. Environ. Pollut..

[37] Rochester JR, Bolden AL (2015). Bisphenol S and F: A systematic review and comparison of the hormonal activity of bisphenol A substitutes. Environ. Health Perspect..

[38] Okai S (2017). Intestinal IgA as a modulator of the gut microbiota. Gut Microbes.

[39] Kurashima Y, Goto Y, Kiyono H (2013). Mucosal innate immune cells regulate both gut homeostasis and intestinal inflammation. Eur. J. Immunol..

[40] Zhao C (2019). Immunotoxic potential of Bisphenol F mediated through lipid signaling pathways on macrophages. Environ. Sci. Technol..

[41] Bollrath J, Powrie FM (2013). Controlling the frontier: Regulatory T-cells and intestinal homeostasis. Semin. Immunol..

[42] Luo S (2016). Gestational and lactational exposure to low-dose bisphenol A increases Th17 cells in mice offspring. Environ. Toxicol. Pharmacol..

[43] Kim KS (2016). Dietary antigens limit mucosal immunity by inducing regulatory T cells in the small intestine. Science.

[44] DeBenedictis B, Guan H, Yang K (2016). Prenatal Exposure to bisphenol A disrupts mouse fetal liver maturation in a sex-specific manner. J. Cell Biochem..

[45] Rosenfeld CS, Trainor BC (2014). Environmental health factors and sexually dimorphic differences in behavioral disruptions. Curr. Environ. Health Rep..

[46] Bauer SM (2012). The effects of maternal exposure to bisphenol A on allergic lung inflammation into adulthood. Toxicol. Sci..

[47] Stromnes IM, Cerretti LM, Liggitt D, Harris RA, Goverman JM (2008). Differential regulation of central nervous system autoimmunity by T(H)1 and T(H)17 cells. Nat. Med..

[48] Yen D (2006). IL-23 is essential for T cell-mediated colitis and promotes inflammation via IL-17 and IL-6. J. Clin. Investig..

[49] Bianchi E, Rogge L (2019). The IL-23/IL-17 pathway in human chronic inflammatory diseases—New insight from genetics and targeted therapies. Microbes Infect..

[50] Biedermann S, Tschudin P, Grob K (2010). Transfer of bisphenol A from thermal printer paper to the skin. Anal. Bioanal. Chem..

[51] Hormann AM (2014). Holding thermal receipt paper and eating food after using hand sanitizer results in high serum bioactive and urine total levels of bisphenol A (BPA). PLoS ONE.

[52] Agnish N (1997). The rationale for culling of rodent litters. Fundam. Appl. Toxicol..

[53] Ilchmann-Diounou H (2019). Early life stress induces type 2 diabetes-like features in ageing mice. Brain Behav. Immun..

