# Supplementary figures and images for: Bisphenol A, S or F mother’s dermal impregnation impairs offspring immune responses in a dose and sex-specific manner in mice
Source: Sci Rep. 2021 Jan 18;11:1650. doi: 10.1038/s41598-021-81231-6 (PMC7813853; doi:10.1038/s41598-021-81231-6)

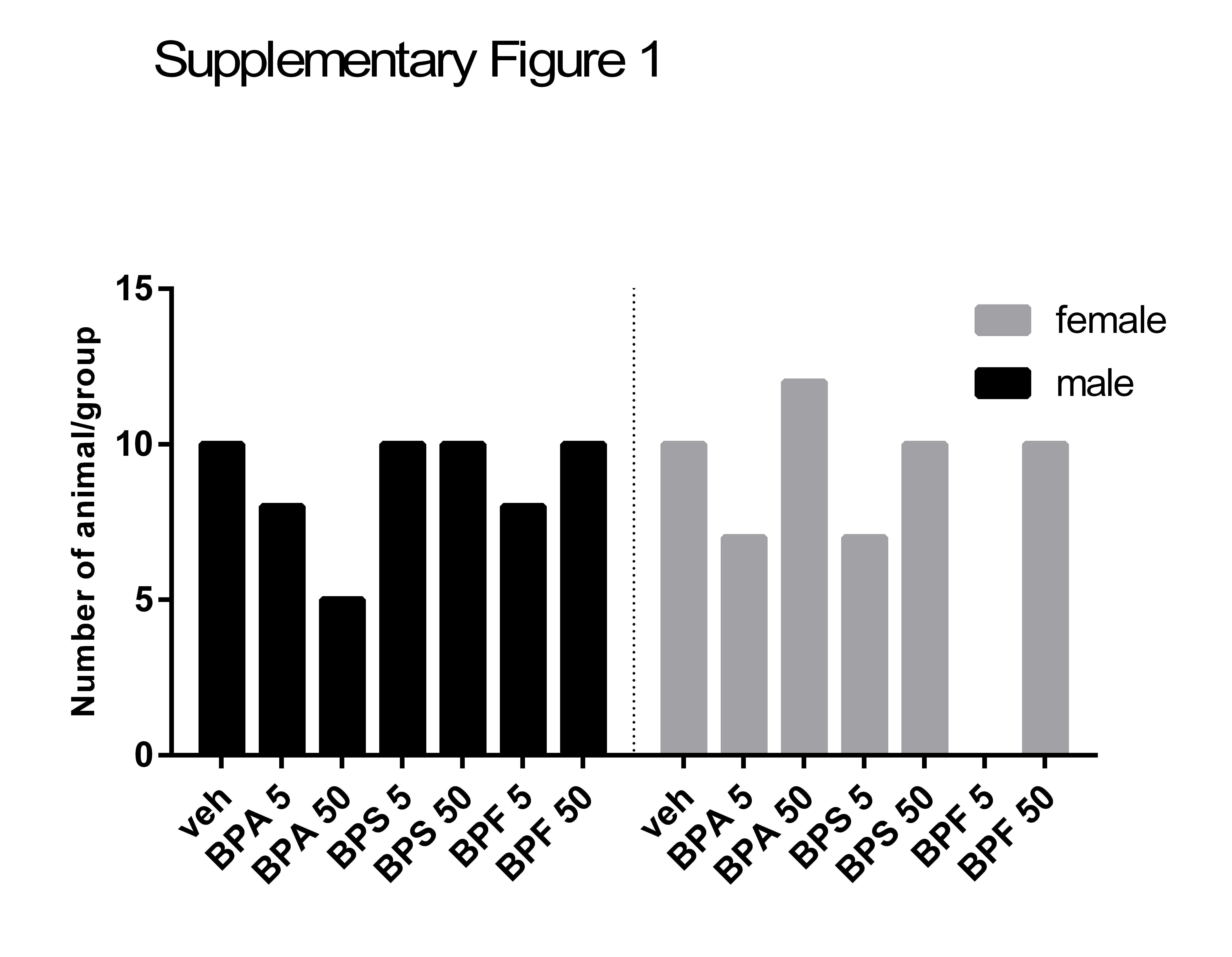

Supplement: Supplementary file 1 — Supplementary Figure 1. [file 41598_2021_81231_MOESM1_ESM.jpg]

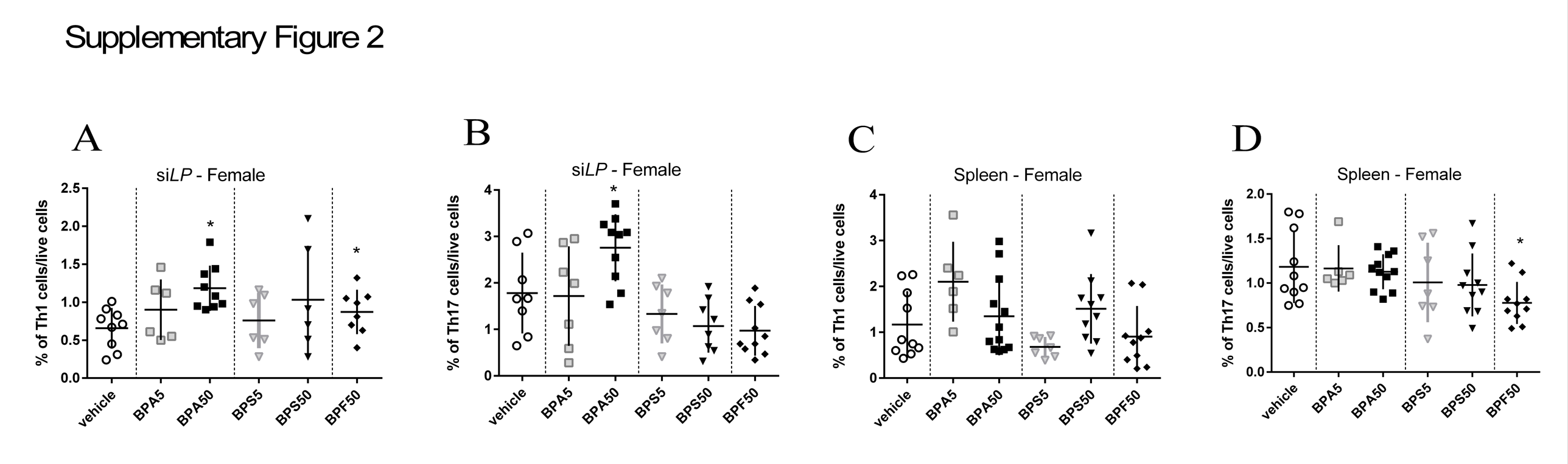

Supplement: Supplementary file 2 — Supplementary Figure 2. [file 41598_2021_81231_MOESM2_ESM.jpg]

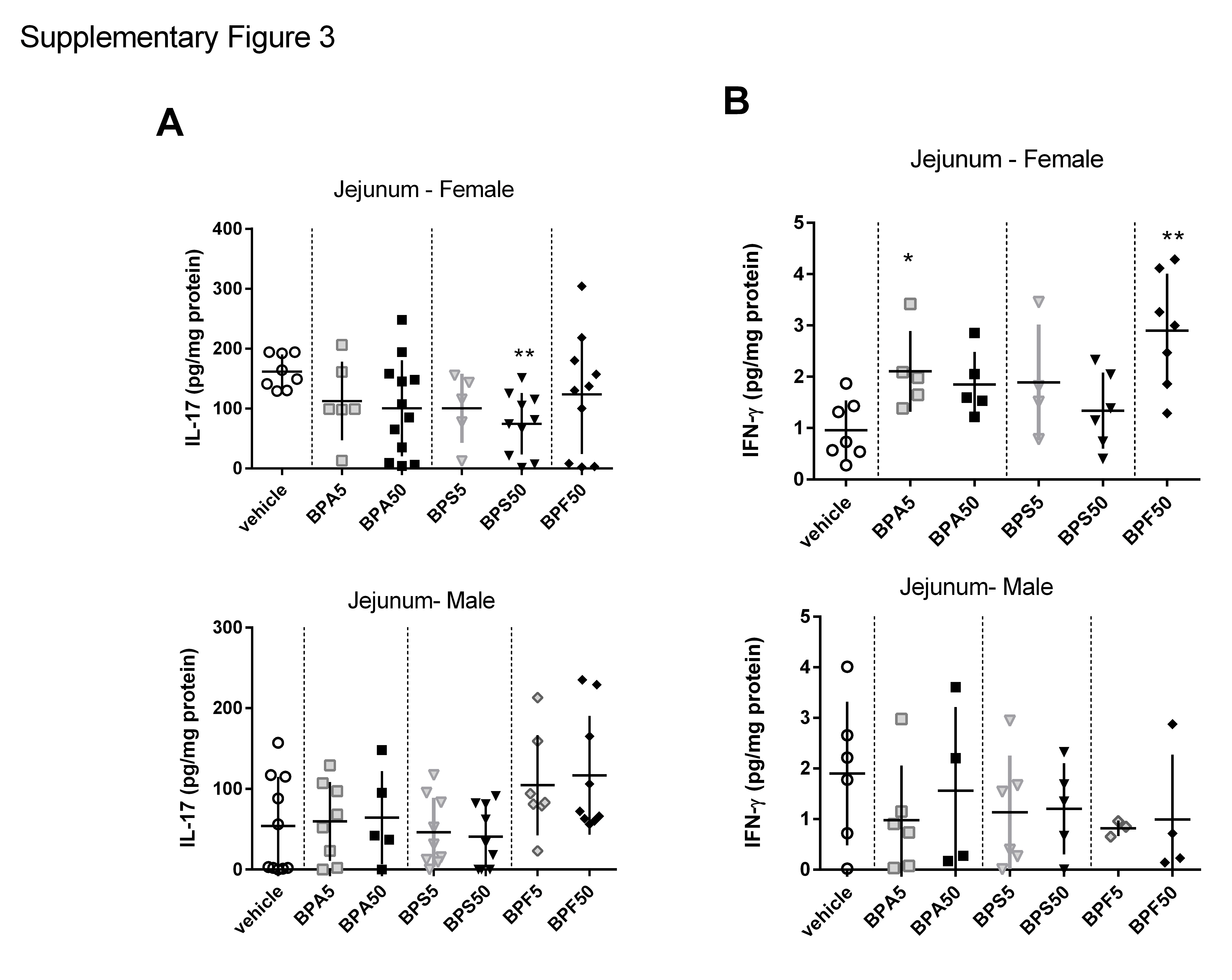

Supplement: Supplementary file 3 — Supplementary Figure 3. [file 41598_2021_81231_MOESM3_ESM.jpg]

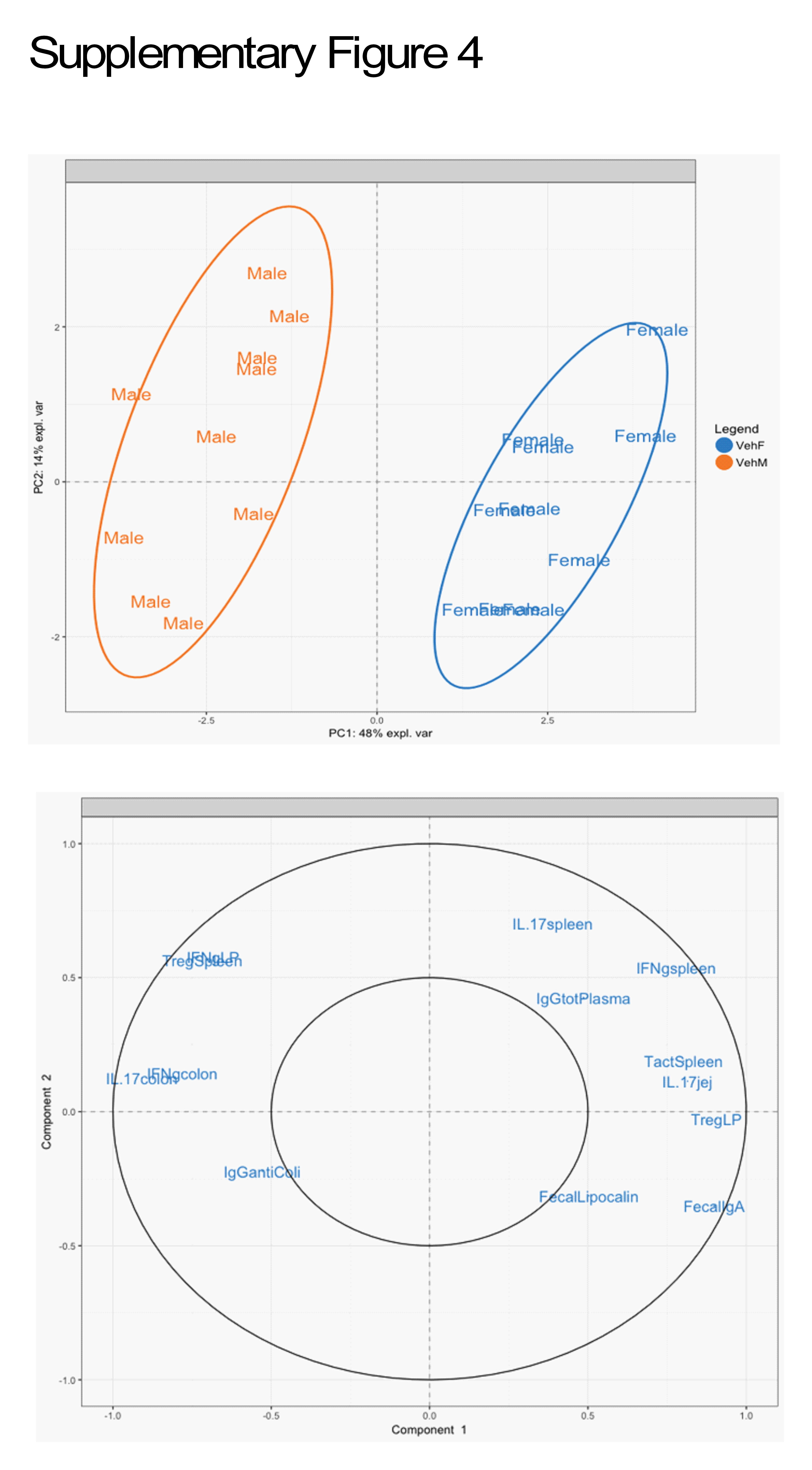

Supplement: Supplementary file 4 — Supplementary Figure 4. [file 41598_2021_81231_MOESM4_ESM.jpg]
