# Supplementary material for: Bisphenol A, S or F mother’s dermal impregnation impairs offspring immune responses in a dose and sex-specific manner in mice
Source: Sci Rep. 2021 Jan 18;11:1650. doi: 10.1038/s41598-021-81231-6 (PMC7813853; doi:10.1038/s41598-021-81231-6)
Supplement: Supplementary file 5 — Supplementary Caption. [file 41598_2021_81231_MOESM5_ESM.pdf]

Bisphenol A, S or F mother's dermal impregnation impairs offspring immune responses in a dose and sex-specific manner in mice

Yann Malaisé, Corinne Lencina, Christel Cartier, Maïwenn Olier, Sandrine Ménard and Laurence Guzylack-Piriou*

Legend

Supplementary data figure

Supplementary Fig 1. Number of offspring mice analyzed/group of treatment.

Supplementary Fig 2. Effects of dermal exposure to bisphenols on Th1/Th17 cell frequency in adult offspring mice

Flow cytometry analysis of Th1: $CD3^{+}IFN\text{-}\gamma^{+}T\text{-}bet^{+}$ (A, C) or Th17: $CD3^{+}ROR\gamma t^{+}IL\text{-}17^{+}$ (B, D) lymphocytes from si*LP* (A, B) or spleen (C, D). * P<0.05 vs. vehicle group. N = 5-12 offspring mice per group.

Supplementary Fig 3. Impact of dermal exposure to BPA on potent IL-17 and IFN-γ inflammation in jejunum of adult offspring mice

IL-17 (A) or IFN-γ (B) in jejunal samples from female and male offspring mice at PND70. * P<0.05 vs. vehicle group. N = 5-12 offspring mice per group.

Supplementary Fig 4. PCA in vehicle group

PCA representation (sample score plot) of gut barrier functions and immune response-associated parameters (loading plot) for control group in male and female offspring mice. Each group are shown in different color. Only loadings with correlation threshold>0.5 were represented on the loading plots. % expl var: percentages for each first two components explained by the model. N = 5-12 offspring mice per group.
